# Patient Reported Outcome Following the Skoog Unilateral Cleft Lip Repair among Adults- a Long-Term Cohort Study and Comparison to a Non-cleft Population

**DOI:** 10.1177/10556656231177139

**Published:** 2023-05-29

**Authors:** Roshan Peroz, Malin Hakelius, Alberto Falk-Delgado, Yun Phua, Maria Mani

**Affiliations:** 1 Department of Plastic and Reconstructive Surgery, Department of Surgical Sciences, Uppsala University and Uppsala university hospital, Uppsala, Sweden; 2 411522 Department of Plastic and Reconstructive Surgery, Karolinska University Hospital, Stockholm, Sweden; 3 Department of Plastic and Reconstructive Surgery, Queensland Children's Hospital, Brisbane, Australia

**Keywords:** lip form, cleft lip and palate, patient satisfaction, craniofacial surgery

## Abstract

**Objective:**

The study primarily aimed to compare satisfaction with lip appearance among adults treated for unilateral cleft lip and palate (UCLP) with Skoog's primary lip repair procedure to those without clefts. The secondary aim was to determine whether satisfaction with lip appearance and the desire to change the lip/face appearance correlated with the number of secondary lip revisions performed.

**Design:**

Long-term follow-up

**Patients/settings:**

All UCLP patients treated at the Uppsala University Hospital born between 1960- and 1987 (n  =  109) were invited. At an average of 37 years following the primary lip repair, the participation rate was 76% (n  =  83). A control group of adults without cleft (n  =  67) completed the same study protocol for comparison.

**Main Outcome Measures:**

Satisfaction with appearance was measured with The Satisfaction with Appearance Questionnaire (SWA) and a modified version of the Body Cathexis -Scale was used to assess the desire to change the lip and facial appearance.

**Results:**

UCLP patients were less satisfied with their lip, face, and overall appearance and reported a greater desire to change the appearance of their lips and face than non-cleft controls (p < 0.001). Dissatisfaction with lip appearance correlated to a greater willingness to change the appearance of the lip and face. No correlation was found between satisfaction with appearance and the number of the previously performed secondary lip revisions.

**Conclusion:**

Adults treated for UCLP are less satisfied with the appearance of their lips compared to the non-cleft population. The number of secondary revisions does not necessarily correlate to greater satisfaction with lip appearance.

## Background

Satisfaction with the appearance is an essential outcome in cleft care and is regarded as crucial for self-confidence, self-esteem, and healthy psychosocial development.^1–5^ In people with repaired cleft-, lip, and palate (CLP), facial aesthetics play a vital role in their quality of life.^6^ With the lips being centrally located on the face, subtle differences can attract attention.^7^ As such, the primary objective of surgical treatment in people with CLP-related facial differences is to normalize the facial appearance by minimizing asymmetry and scarring.^8^ Many adults treated for CLP, however, still show some stigmata following cleft lip repair, which affect the facial appearance and attract greater attention than a face without cleft.^9–11^

There are many surgical techniques for lip repair.^12–19^ While each technique has certain purported advantages and disadvantages, few studies have evaluated the long-term outcomes of these techniques.^20–26^ Despite advances in treatment, patients treated for CLP express concerns regarding the function, shape, and appearance of the lip as well as the nose.^27–29^

Skoog lip repair has been the technique of choice at our institution for the last 60 years.^17^ The Skoog lip repair experience in Uppsala for UCLP deformity has previously been published by Falk-Delgado et al.^30^ The study by Falk-Delgado and co-workers reported complications after lip surgery and the incidence and indication for lip revision.^30^ Other studies that report outcomes of this technique focus mainly on the nasal or nasolabial appearance.^31^ No previous research has looked at patient-reported satisfaction with lip appearance among adults treated with Skoog's lip repair in infancy.

The current study aimed to assess patient-reported satisfaction with lip appearance in adults treated for UCLP with Skoog's lip technique and compare their self-assessment of lip appearance with that of a matched non-cleft population. The secondary aim was to assess whether satisfaction with lip appearance and the desire to change lip/face appearance correlated with the number of secondary lip revisions performed.

## Material, Patients, and Methods

### Subjects

**Figure 1.**
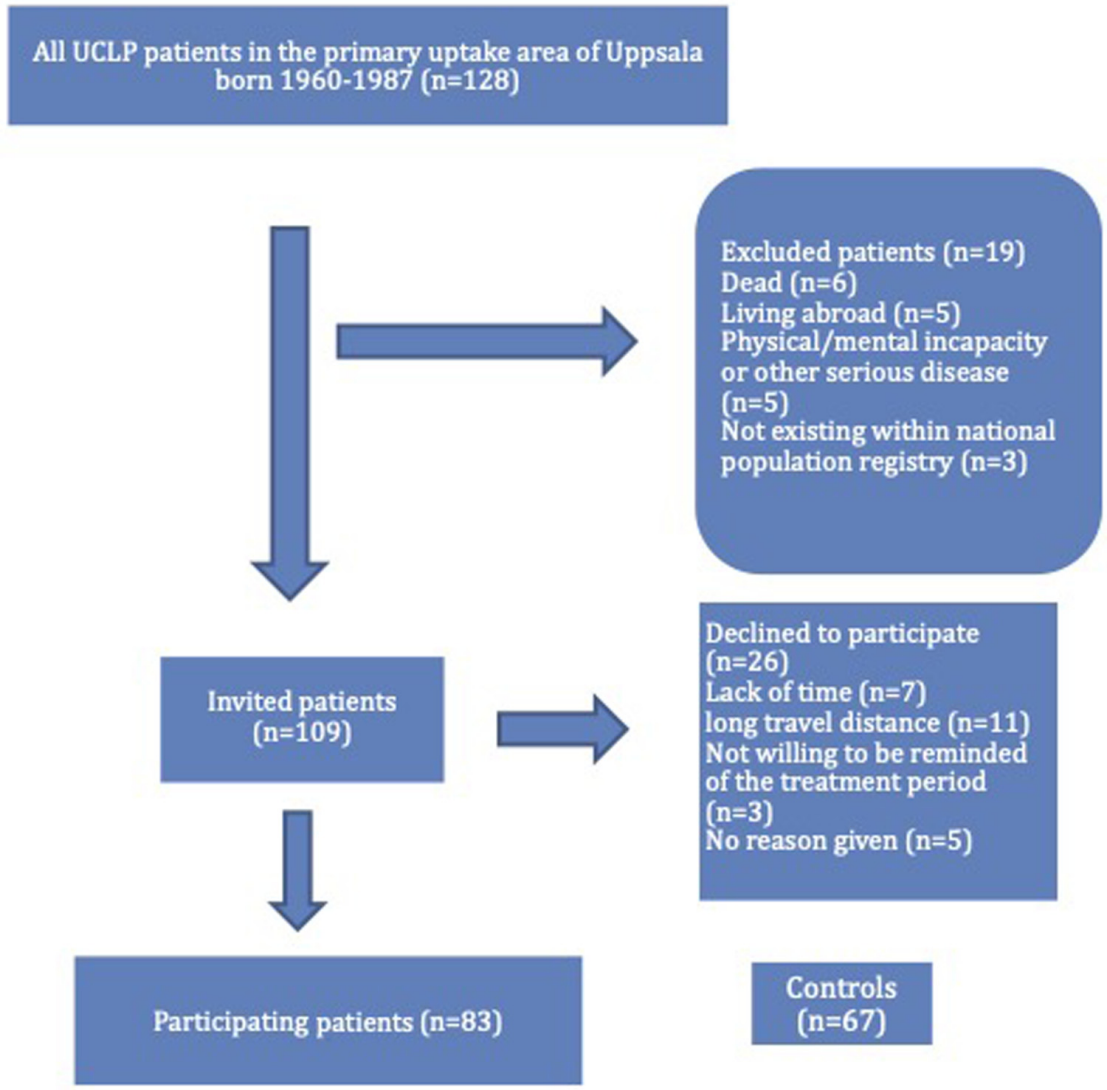
Study population (n  =  number of individuals).

All patients born between 1960 and 1987, with complete UCLP without associated malformations and syndromes, all treated according to standard protocols used at the Department of Plastic Surgery, Uppsala University Hospital (UU), were considered for this study (n  =  128). The Uppsala University Hospital serves about 1.5 million people in its geographical region, with no other hospitals in the region offering care for people with CLP. Out of the 128 patients considered for this study, nineteen were excluded because they had severe illnesses (physical or mental incapability) (Figure 1). A total of 109 patients were invited by an invitation letter, followed by a phone call with further information about the study. At a mean of 37 years following the first primary lip repair, 76% (n  =  83) participated. The reasons given for non-participation can be seen in (Figure 1). There were no differences in age and gender distribution between the participating and non-participating patients. Participants were provided questionnaires for the current study to complete at home following their acceptance to participate. A control group without cleft (n  =  67), drafted from close contacts, staff, and students completed the same study protocol. The age and sex distribution of patients and controls are presented in Table 1.

**Table 1.** Study Population Characteristics.

Variable	All Patient *(*n *=* *83)*	Controls *(*n *=* *67)*
Male n*, (%)*	46 (54)	28 (42)
Female n*, (%)*	38 (46)	39 (58)
Age in years, mean (SD)	34 (7)	33 (8)
Male	33 (7)	32 (6)
Female	35 (7)	33 (10)

n *=* number of individuals, SD = Standard Deviation.

Patients included in the current study had been treated per a specific treatment protocol, including lip closure at three to six months of age with Skoog's lip repair technique^17^ followed by palate closure either by one- or two-stage closure, depending on the time period of treatment. Patients born between 1960 and 1975 were treated using a one-stage technique first reported by Veau and Wardill and later modified by Skoog at an average age of 1.9 years,^32–34^ whereas those born 1976-1987 were treated according to the two-staged technique.^35–37^

None of the patients had, primary nose surgery performed. Until adolescence, minimal or no surgery on the nose was performed. Secondary surgery on the lip and nose, as well as pharyngoplasty, were performed based on the patients’ specific needs. Mani and co-workers has previously described the study protocol in detail.^38^ Data on previous surgery and the number of secondary lip revisions were retrieved from the patients’ clinical records.

### Self-Assessment of Appearance

To evaluate satisfaction with lip and facial appearance items from, two different questionnaires were used: the satisfaction with appearance scale (SWA) and the modified version of the body cathexis -scale.^39–41^

The SWA was developed by the Psychology Special Interest Group of the Craniofacial Society of Great Britain and Ireland.^39^ The questionnaire covers aspects related to the appearance of the different facial areas, general appearance, and speech. The questionnaire has previously been used in Scandinavia, and a Swedish language version was used for this study.^31,42^ The three components from the SWA included in the analysis asked for a self-assessment of the patient's overall appearance, face, and lips. Questions included in the current study were: “*What do you think about your appearance (overall impression),” “What do you think of your face?” and “what do you think of these parts of your face? Lips*” Visual Analogue Scale (VAS) was used to answer the questions with markings along a line ranging from 0 to 10, with 0 indicating a very high level of satisfaction and 10 indicating a low level of satisfaction. A test panel completed the questionnaire for comprehensiveness and relevance before the study.

The modified version of the body cathexis score used as by Marcusson and co-workers^40,41^ consists of 22 items related to facial appearance, desire for further treatment, and speech function. In this study, only questions regarding the desire for further treatment to change the facial appearance and the appearance of the lips were included. The questions included were: “If it were possible, would you like to change your facial appearance?” and “If it would be possible, would you like to change the appearance of your lips?”. These questions were also answered on a (VAS) from 0 to 10. Where 0 indicates “not at all” and 10 indicate “very much .” The complete questionnaire has previously been tested for reliability by Marcusson et al.^43^

### Statistical Analysis

The data were tested for normality using the Shapiro-Wilks test. Mann-Whitney U test compared numeric data from the VAS due to skewed distribution. A p-value of <0.05 was considered statistically significant. The correlation was made by Spearman's rank test (p < 0.05). The computer software for statistical analysis, IBM SPSS 24.0 (IBM corporation, Somers, NY), was used for all the analysis.

### Ethics Considerations

The study was granted approval from Regional Research Ethics Committee (Reference number 2005:245). All participants in the current study gave their informed consent.

## Results

Population characteristics are presented in Table 1.

**Figure 2.**
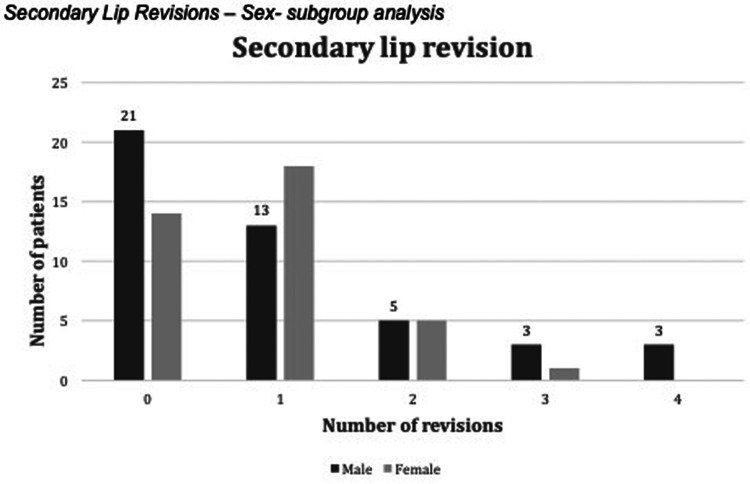
Number of lip revisions based on sex.

Secondary lip revisions were performed in 48 patients - (58%). The median number of secondary lip corrections was 1 per patient (range 1 to 4). Seventeen subjects had more than one lip revision. Out of the 48 patients who underwent secondary lip revision, 31 underwent one lip revision, 10 underwent two lip revisions, 4 underwent three lip revisions, and 3 underwent four revisions. No difference was seen in secondary lip revisions based on sex. Rhinoplasty was performed in 65 patients (78%). The number of secondary lip revisions based on sex is presented in (Figure 2).

## Satisfaction with the Appearance

### Comparison of all Patients to all Controls

**Figure 3.**
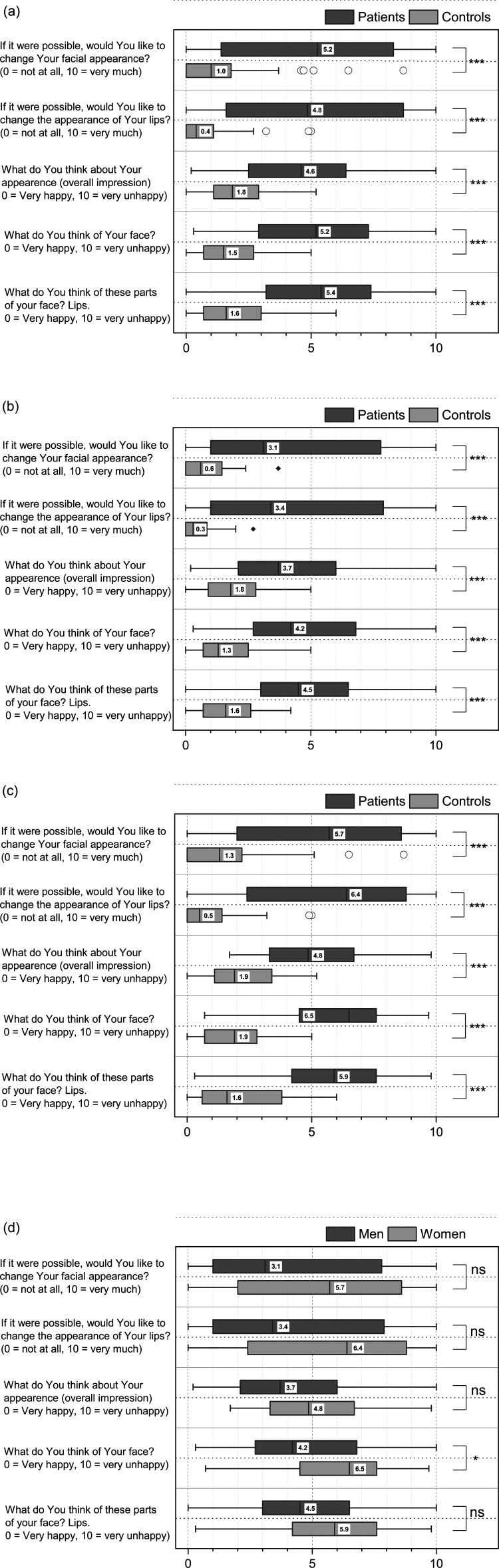
a. Comparison of all patients and controls. b. Male subjects with UCLP compared to male controls. c. Female subjects with UCLP compared to female controls. d. Female subjects compared to male subjects.

Patients rated lower satisfaction with lip and facial appearance and overall appearance than controls (p < 0.001). Additionally, patients also expressed a greater desire to change the appearance of their lips and face than the control group (p < 0.001) (Figure 3a).

### Sex- Subgroup Analysis

Both male and female patients in a subgroup analysis were less satisfied with their lip and facial appearance as well as their overall appearance compared with controls (p < 0.001). Both groups also reported a greater desire to change the appearance of the lips and face (p < 0.001) (Figure 3b-c). When female UCLP patients were compared to male ULCP patients, they rated lower for satisfaction with facial appearance (p < 0.05). No other differences were found between the ratings by female and male patients (Figure 3d).

## Satisfaction with the Appearance and Number of Revision Surgeries

Dissatisfaction with lip appearance correlated with a higher desire to change the appearance of the lips; Rho (Spearman's rank correlation coefficient) was (0.713, p < 0.001) and facial appearance (0.602) among patients with UCLP (Table 2). The same correlation was found when controls without cleft were analyzed separately. The number of previous secondary lip revisions correlated to a higher desire to change lip appearance (0.316) and facial appearance (0.327). However, no significant association was found between the amount of previous secondary lip revisions and satisfaction with lip appearance Rho; (0.232) and facial appearance (0.186) (Table 2).

**Table 2.** Association Between Satisfaction of lip Appearance and Number of lip Corrections and Desire to Change the Appearance of Lip and Face.

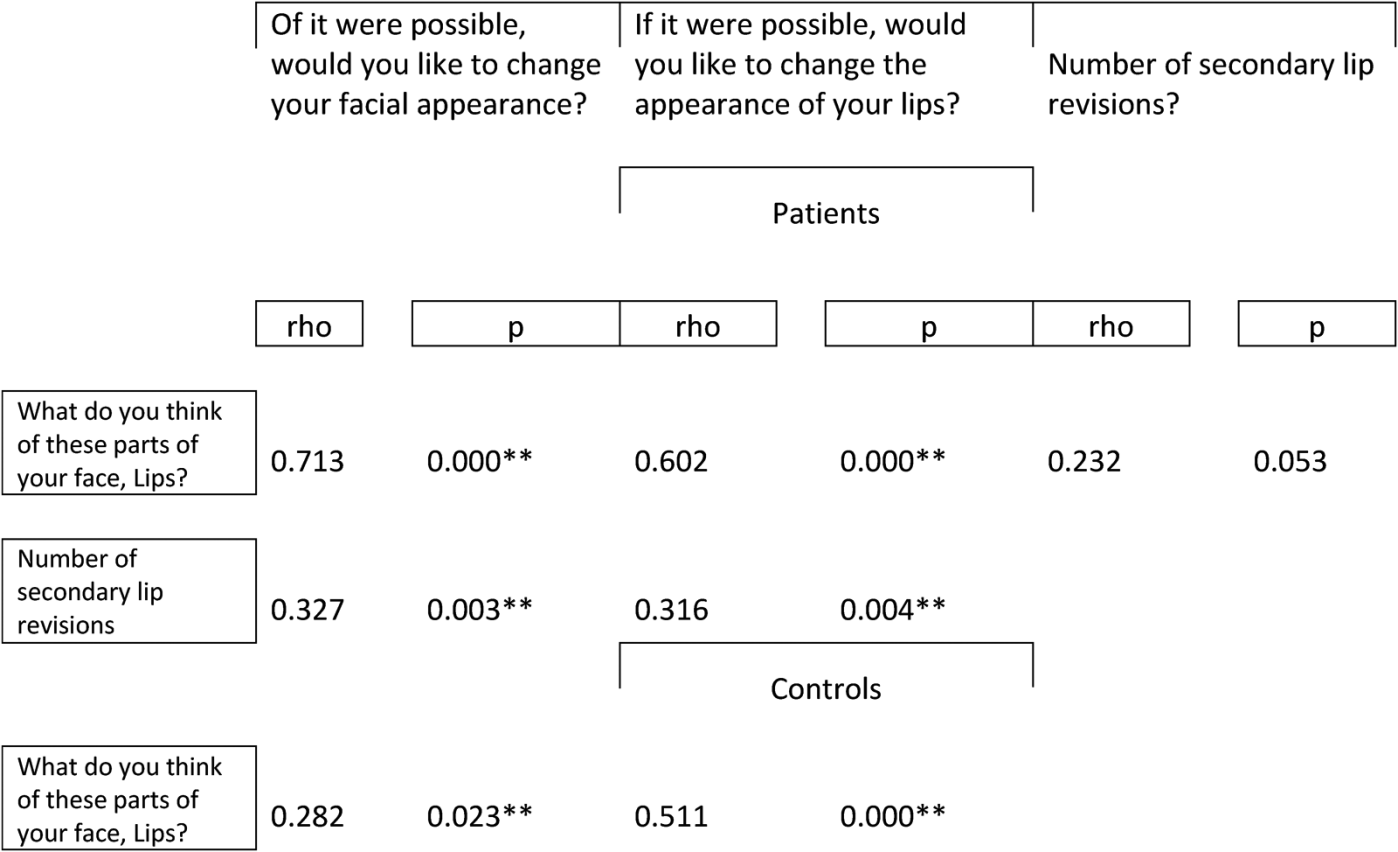

rho = Spearman's rank correlation coefficient, Significant correlation is marked with bold (*P < 0.05, **p < 0.01).

## Discussion

To our knowledge, no earlier studies evaluating long-term patient satisfaction with lip appearance following Skoog's technique of lip repair have been published. This study is unique in that it had a response rate of 76 percent and a mean follow-up length of 37 years. In addition, only four different physicians performed 95% of all surgical procedures. The patients in this study were given treatment following specified protocols for surgeries, evaluations, and records, which have been kept alike since 1960. This includes regular follow-ups by a team of professionals involved in treating CLP patients, such as therapists, orthodontists, phoneticians, and plastic surgeons. This uniformity of our series of patients allows the long-term evaluation of the self-reported assessment of lip appearance.

Patient satisfaction with facial appearance is an important outcome measure in the treatment of CLP as well as for cleft care improvement (Johnson & Sandy, 2003; Urden, 2002). Various quantitative and qualitative methods have been developed to assess facial and nasolabial appearance. Quantitative studies use anthropometric, automatic, or manual measurements to quantify the morphology and symmetry of the lips and nose on two- or three-dimensional photographs.^44–50^ Qualitative studies, on the other hand, focus on subjective assessment by scoring facial appearance, using different assessment tools, designs, and combinations of assessors.^51–53^ One subjective method of nasolabial appearance assessment is the index developed by Asher-McDade et al.^54^ This index has been used in several studies, such as the Eurocleft study,^55^ the follow-up Eurocleft study^56^ and the Clinical Standards Advisory Group (CSAG study).^57^ Other subjective measures include patient-reported outcome measures such as the reliable and validated CLEFT-Q.^58^ The current study uses two different patient-reported outcome measures. The SWA is used to determine how satisfied patients are with their lip and face appearance^39^ and the modified version of the body cathexis score^41,43^ to assess the desire to change the lip and facial appearance.

The current study found that adults with UCLP were less satisfied with the appearance of their lips, face, and overall appearance compared to controls without UCLP. Furthermore, patients with UCLP reported a greater desire to change the appearance of their lips and face compared to controls. Similar results were found when subgroups based on sex were analyzed separately.

These findings are in accordance with previous studies demonstrating generally unfavorable appearance ratings by cleft patients.^40,59^ Oosterkamp and co-workers found that patients with bilateral CLP were more dissatisfied with the nose and upper lip appearance than controls without a cleft.^29^ Similarly, patients with CLP have reported greater dissatisfaction with their appearance than their parents^1,60^ and medical professionals.^40^ Cleft patients have also been shown to rate their nose/upper lip worse than their face.^2,11^ A previous study by Mani and co-workers showed that subjects with UCLP malformation and controls without cleft did not differ in the self-assessment of the non-cleft features like hair and ear, indicating similar body image perception. However, when it came to cleft-related features such as nose, lip, and face, people with UCLP were more negatively affected.^31^ Although these studies did not focus on the appearance of the lip and face but rather on the nasolabial appearance, they all confirmed that people with CLP malformations are more dissatisfied with the features related to the cleft malformation compared to people without CLP malformation, which agrees with the findings of the current study.

Our results showed that women with UCLP were more unhappy with their facial appearance than men, though they did not differ in their self-assessment of the lip, overall appearance, or desire to change the appearance. Previous studies have found similar satisfaction levels between males and females; however, the included patients were younger (aged 8–21 years).^61^ Similarly, no difference in appearance satisfaction was found between males and females with bilateral CLP.^29^ However, a study from Sweden found that the mouth and the face profile ratings were poorer among females with CLP than in men.^40^ Differences in the subgroup analysis based on gender in this study for facial appearance may be explained by the different perceptions of attractiveness and importance of beauty in society for men and women. According to Kapp et al., women may be more critical of their appearance than men due to a greater emphasis placed on physical attractiveness in females by society (Kapp, 1979), which may explain why facial appearance was overall rated more poorly by females cleft patients compared to males in the current study. However, this does not explain why other parameters related to appearance were similar between males and females.

Patients may undergo corrective procedures following primary repair of the cleft defect later in adolescence of secondary deformities aiming to restore lip and nose symmetry. In our study, fifty-eight percent of patients with UCLP underwent at least one lip revision surgery, with 17 patients having more than one lip corrective surgery. On average, patients with UCLP underwent one secondary lip correction. Additionally, 78% of UCLP patients underwent at least one rhinoplasty. The revision frequency is in accordance with previous reports. A 25 - year follow-up study on patients treated with Millard's technique for isolated unilateral cleft lip reported that 80% of the included patients underwent at least one corrective lip surgery, with 37% having more than one.^20^ However, even if the study by Becker et al. reports on the rate of secondary revisions of complete and partial isolated cleft lip, it still indicates the frequency of corrective revisions. Our results show that patients operated with Skoog's lip repair technique are associated with a total revision rate comparable with other techniques.^20^ Similar findings were previously published by Falk-Delgado and co-workers, who found that Skoog lip repair is related to a low total revision rate in patients with UCLP, unilateral cleft lip, and cleft lip and alveolus born 1960–2004.^30^ However, the goal of this study was not to evaluate the surgical technique and total revision rate but rather to report on the satisfaction of lip appearance among adults treated with the Skoogs technique compared to non-cleft controls.

Dissatisfaction with lip appearance was correlated to a higher desire to change the lip and facial appearance. Despite this, the number of secondary lip corrections did not correlate to the satisfaction of lip and facial appearance. These findings are in accordance with results in previous studies in patients with varying clefts.^1,28,40,59^ Surprisingly, the results of this study showed that despite numerous surgical lip revisions, patients still expressed a greater desire to change the appearance of the lip and face. Indicating that patients still felt compromised by their appearance despite the number of lip revisions. However, caution should be used when drawing conclusions because it's hard to tell if the desire to improve the lip and face appearance or other consequences, such as initial expectations, cause more lip revisions.

Various factors may limit the accuracy of self-assessment of appearance and satisfaction with the outcome after cleft lip and palate treatment. A previous study reported that patients feel grateful to their cleft surgeon and may not discuss dissatisfaction with facial features related to the CLP.^1,40^ This may lead to an under-reporting of cleft-related appearance concerns. An important factor influencing patient satisfaction is their expectations.^62,63^ Satisfaction is related to how the perception of treatment meets patients’ expectations. Subjects with lower expectations tend to be more satisfied.^62^ Unrealistic expectations regarding the outcome of surgical treatment may anticipate poor psychosocial outcomes.^64^ Realistic and honest information about what can and cannot be achieved in cleft care may help to generate more realistic expectations, resulting in less unhappiness with one's appearance.

The surgical technique and surgeon's experience may be essential in the success of the outcomes, but the extent of the primary cleft malformation, width, underdevelopment of tissues and the misplacement of structures may also influence the reconstructive result. A previous study on the same study population showed that a larger posterior cleft width measure is associated with less satisfaction with nasal appearance.^6^ Both qualitative studies with different combinations of assessors and, quantitative studies are required in order to assess the true effectiveness of a surgical technique.

The age range of the participants in this study could be a potential drawback (20–47 years). It is important to note, however, that all participants were adults with complete growth of the face, which is preferable for assessing the appearance as the growth of the face is an unpredictable factor that can affect the final outcome.^20^ Despite the fact that this study only covered a small number of patients, the participation rate of 76 percent, 37 years after the first surgery, is highly unique in this field. Another limitation of this study is attributed to the fact that different self-assessment questionnaires were employed. However, both questionnaires used the same scale (VAS), making the comparison easier, although not ideal. The SWA questionnaire has similarities to the Cleft evaluation profile instrument developed by the Royal College of Surgeons cleft lip and palate audit group.^1,2^ The SWA questionnaire's internal consistency and coherent factors were found to be adequate.^39^ The questionnaire has previously been used in Scandinavia.^31,42^ The questionnaire (Body Cathexis Score) assessing the desire to change the appearance of the lip and face has previously been tested for reliability by Marcusson et al.^43^ Another limitation of the current study concerns the selection bias of questions included in this study from two different complete questionnaires. Due to selection bias and neglect, important aspects and factors related to appearance could have been missed. However, the study's goal was not to evaluate factors that can affect and are associated with appearance but rather to explore satisfaction with lip appearance and the correlation between satisfaction/desire to change the lip appearance and the number of secondary lip revisions. Additionally, no validated lip questionnaires existed at the time of the study. Another drawback attributed to the questionnaires is that they do not capture the whole picture related to the satisfaction of appearance, such as self-perception and realistic expectations, which have been proven to play a significant role in self-satisfaction assessment, as discussed and described earlier.

The current study registered questions on a VAS that uses a continuous scale, allowing for easy conversion of qualitative assessment into metric values. Visual analogous scale (VAS) has been shown to be more repeatable, objective, sensitive, and reliable than categorical scales.^65–68^ A disadvantage with the VAS scale is, however, attributed to the fact that data is not distributed normally in all cases, and therefore it cannot connect to numbers in ordered scales and categories.^69^

## Conclusion

Adults with UCLP treated with the Skoog procedure as a child are less satisfied with the appearance of their lips compared to a non-cleft population. The number of secondary revisions does not necessarily correlate to greater satisfaction with lip appearance.
